# Varicella Skin Complications in Childhood: A Case Series and a Systematic Review of the Literature

**DOI:** 10.3390/ijms17050688

**Published:** 2016-05-06

**Authors:** Elena Bozzola, Mauro Bozzola, Andrzej Krzysztofiak, Alberto Eugenio Tozzi, May El Hachem, Alberto Villani

**Affiliations:** 1 Department of Pediatrics, Pediatric and Infectious Diseases Unit, Bambino Gesù Children’s Hospital, IRCCS, Piazza Sant’Onofrio 4, 00165 Rome, Italy; krzy@opbg.net (A.K.); alberto.villani@opbg.net (A.V.); 2 Internal Medicine and Therapeutics Department, Pediatrics and Adolescentology Unit, University of Pavia, Fondazione IRCCS San Matteo, Viale Golgi 19, 27100 Pavia, Italy; mauro.bozzola@unipv.it; 3 Multifactorial Disease and Complex Phenotype Research Area, Bambino Gesù Children’s Hospital, IRCCS, Piazza Sant’Onofrio 4, 00165 Rome, Italy; albertoeugenio.tozzi@opbg.net; 4 Department of Pediatrics, Dermatologocical Unit, Bambino Gesù Children’s Hospital, IRCCS, Piazza Sant’Onofrio 4, 00165 Rome, Italy; may.elhachem@opbg.net

**Keywords:** varicella, children, skin complication, hospitalization

## Abstract

Even if varicella is generally considered a harmless disease in childhood, severe complications may occur. We examined varicella skin complications (VSCs) in hospitalized immunologically healthy children, over a nine-year period. We also systematically analyzed previous reports to calculate the rate of VSCs in the literature. VSCs occurred in 16.4% of children hospitalized for varicella. This figure is in accordance with the literature, as the range of VSCs was 2.6%–41.2%. Skin complications may represent determinants of hospitalization and of other indirect costs in young children.

## 1. Introduction

Varicella is one of the most common infectious exanthematous diseases, with a worldwide distribution and mostly affecting patients at pediatric age. Even if it is generally a benign self-limiting disease, it can lead to severe complications that may require hospitalization, such as bacterial infection, respiratory impairment, and neurological deficits. Patients with a history of underlying malignancy, immunosuppressive therapy, or organ transplantation are susceptible for a severe varicella course due to impaired cellular immunity. However, immunocompetent children may also require hospitalization due to a complicated course [1,2,3,4].

Varicella skin complications (VSCs) may include cellulitis, abscess, necrotizing fasciitis, impetigo, and gangrene. In the literature, the rates of VSCs range from 2.6% to 41.2% in the pediatric population [4,5].

## 2. The Aim of the Study

The aim of the study is to review the type and the rate of VSCs in immunologically healthy pediatric patients hospitalized for varicella at Bambino Gesù Children’s Hospital (OPBG), Roma, Italy. We also analyzed the literature to calculate the rate of VSCs among the pediatric population.

## 3. Results

### 3.1. Varicella Skin Complications (VSCs)

Among all the children hospitalized in the study period, 431 were admitted for varicella. The mean age of patients was 3.19 years (ranging from 2 weeks to 17.5 years). Males (57.4%) were not significantly more affected than females (42.6%).

**Figure 1:**
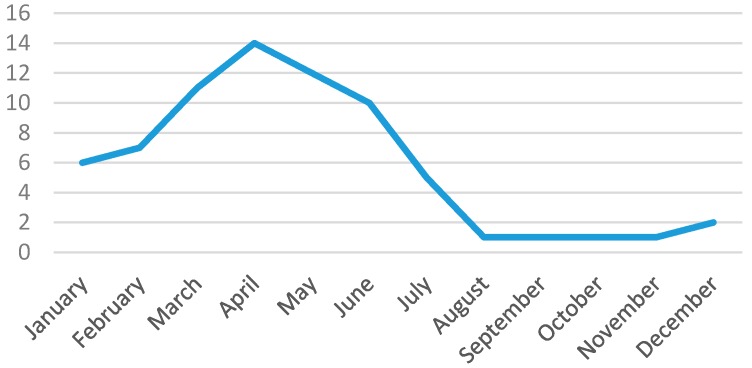
Monthly incidence of hospitalized varicella skin complications.

Out of 431 patients, 71 presented with VSCs (16.4%; 95% CI: 13.0%–20.0%). VSCs had a seasonal distribution with a peak of incidence in spring, as reported in Figure 1.

Some patients had more than one skin infection. The most frequent complications were impetigo in 50 patients (70.4%), cellulitis in 20 (28%), and abscess in 5 (7%). No cases of fasciitis, scarlet fever, staphylococcal scalded skin syndrome, varicella gangrenosa, or felon were recorded. In 76% of children, the VSCs had been the only reason for hospitalization. As for the others, we found neurological (1.4%), pulmonary (4.2%), upper respiratory (8.4%), and gastrointestinal (4.2%) complications. Four patients presented with a systemic bacterial superinfection. Patients with VSCs were aged 2.67 years (ranging from 60 days to 10.5 years). Most cases occurred in children aged 1 to 5 years (53.5%). As for the others, 21 (29.5%) were younger than 1 year, 11 (15.5%) were aged 5–10 years, and just 1 was older than 10 years. All patients underwent a blood examination to test immunoglobulin and T subset cell count, which proved normal. No children had previously received varicella vaccination. Males (52.2%) were slightly more affected than females (47.8%). The median hospitalization was 8.5 days. All children were intravenously prescribed an antibiotic treatment to prevent bacterial skin infection. Fifty-four patients (76%) were also treated with intravenous acyclovir. No sequelae were expected at discharge, and no patients died.

### 3.2. Literature Review

Out of 259 reports resulting from our MEDLINE analysis, 18 manuscripts matched the criteria for inclusion in the meta-analysis. They were carefully examined to estimate a pooled rate of VSCs [2,3,4,5,6,7,8,9,10,11,12,13,14,15,16,17,18,19]. The other 241 manuscripts were excluded because they: (1) did not report the precise number of VSCs in a precise period (132); (2) concerned only children with immunodeficit or with malignancy (9); (3) were case reports (71); (4) concerned adults (5); (5) reported indistinguishable data on the adult and pediatric populations (2); (6) reported indistinguishable data on both hospitalized and treated-at-home children (5); (7) concerned just one specific VSC (13); (8) had overly strict inclusion criteria (3) (one manuscript concerned only children younger than twelve months, one report just previously vaccinated patients, and one report only skin infections complicating congenital varicella syndrome). Finally, one study was excluded because the same data were reported in a previous report by the same author.

**Figure 2:**
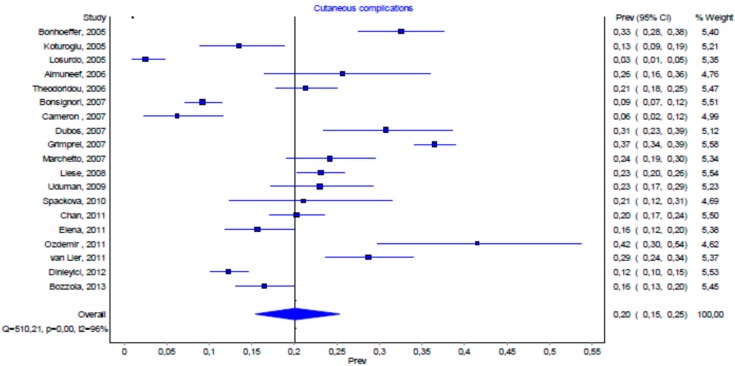
Prevalence of varicella skin complications (VSCs) in hospitalized patients (the % weight of the studies is calculated on the inverse of the variance by using the DerSimonian and Laird method).

Figure 2 synthesized the pooled prevalence of VSCs. We found a substantial heterogeneity among the studies in the systematic review (*p* < 0.001).

## 4. Discussion

Varicella is common among the population. In fact, the mean number of varicella cases in Roma from 2003 to 2013 was, each year, 2235 cases (ranging from 1176 to 3681 cases). Even if varicella has a generally benign course, it may become complicated. Bacterial superinfection of skin lesions is known to occur mostly in infants and toddlers and can cause disfiguring scars. Skin with varicella lesions is likely the portal of entry of either *Staphylococus aureus* or Group A Streptococcus. Indeed, infants represent a vulnerable risk group for developing invasive skin complications, particularly during the first two weeks of disease. Most often, hospitalization is not needed. Nevertheless, in the event of severe infection, varicella can become complicated by cellulitis, abscess, or fasciitis and can require hospitalization.

We explored the rate of VSCs in both children with varicella hospitalized at the Bambino Gesù Children’s Hospital and in those reported in the literature. To the best of our knowledge, no other comparative incidence figures of VSCs have been reported. We found a prevalence of VSCs of 16.4% (CI 13.0%–20.0%) among all those hospitalized for varicella. Our data are in accordance with those described by other authors included in the review of the literature, as the prevalence lies within the CI of 15.0%–25.0% (Figure 1). The difference in the prevalence of VSCs among individual reports, ranging from 2.6% to 41.2%, may be influenced by the patients included in the studies, which presented numerous variables, such as age and the presence of underlying diseases. The methodology of the published reports may also influence the variability of VSCs. In fact, the studies in the meta-analysis used different inclusion and exclusion criteria, such as the age of patients and the presence of underlying diseases [3,7,19]. Differences in the rate of VSC hospitalization may also be correlated to both the sociodemographic characteristic of populations and to the availability of outpatient skin-infection therapy by medical doctors. The different varicella-related hospitalization figures due to skin complication rates may also be correlated to different hospitalization policies. A high rate of hospitalized VSC cases may be connected to the tendency of pediatricians to hospitalize young children in order to prescribe either antibiotic or antiviral intravenous treatment. We observed that, apart from VSCs, cutaneous superinfection was the leading reason for hospitalization. This is in accordance with previous studies in which impetigo was also the first cause of hospital admission [10,16].

## 5. Materials and Methods

We retrospectively included in the study patients younger than eighteen years old admitted to the OPBG for varicella over a nine-year period (from January 2004 to January 2013). The parents of all the subjects gave their informed consent for inclusion in the study. The study was conducted in accordance with the Declaration of Helsinki.

In all patients, varicella was clinically diagnosed. Patients with a congenital or an acquired immunodeficit as well as those affected by malignancy were excluded. We defined a VSC as a cutaneous complication occurring within two weeks of the onset of chickenpox disease and to which the infection may have contributed. Definitions of secondary bacterial skin complications were taken from textbooks and dictionaries.

We made a MEDLINE search to calculate the incidence of VSCs in the literature. For the purpose of this study, we used the keywords “varicella” and “complications” (Subheading) as MESH terms. The search concerned English publications referring to patients younger than 18 years of age, from January 2003 to January 2013. We included in the search: (a) cases of pediatric chickenpox; (b) reported case definitions for VSCs; and (c) VSCs published with precise numerators and denominators. We excluded reports: (1) if they did not report the precise number of VSCs in a precise period; (2) if they concerned only children with immunodeficit or with an underlying disease; (3) if they concerned adults; (4) if they presented undistinguished data on both adults and children; (5) if they included the entire pediatric population and not just hospitalized children; and (6) if they concerned only one specific VSC.

From all reports, we extracted both the number of varicella cases and the number of cases with VSCs. We pooled the estimates of rates and their 95% confidence intervals (CI) by using standard meta-analytic techniques. Data were analyzed using Metanalysis 3, and a pooled estimate of VSCs incidence was calculated by using a random-effects model with inverse-variance weighting by using the DerSimonian and Laird method. A chi-square test for heterogeneity was used to measure statistical heterogeneity.

## 6. Conclusions

Epidemiological studies on the rate of VSCs in the pediatric population are essential for the implementation of appropriate immunization recommendations.

As previous literature has done, we verified that VSCs may also require hospitalization in previously healthy children. Our results may contribute to the development of health policies and immunization strategies. In fact, chickenpox affects not only children but also society, in terms of medical therapy costs and missing work days by parents. A limitation of our study is that a direct comparison with previous reports was often imprecise due to different inclusion and exclusion criteria.
